# Case Report: Simultaneous penetrating keratoplasty with autologous simple limbal epithelial transplantation as an alternative to keratoprosthesis

**DOI:** 10.12688/f1000research.133637.3

**Published:** 2023-07-20

**Authors:** Supriya Sharma, Swati Singh, Swapna S. Shanbhag

**Affiliations:** 1 Cornea and Anterior Segment Services, Shantilal Shanghvi Eye Institute, Mumbai, Maharashtra, India; 2 Shantilal Shanghvi Cornea Institute, LV Prasad Eye Institute, Hyderabad, Telangana, India; 3 Ophthalmic Plastic Surgery Services, LV Prasad Eye Institute, Hyderabad, Telangana, India

**Keywords:** ocular burn, limbal stem cell deficiency (LSCD), limbal stem cell transplantation (LSCT), cicatricial entropion, descemetocele, penetrating keratoplasty

## Abstract

**Introduction and importance**: This case report highlights the multidisciplinary approach required to achieve successful anatomical and functional outcomes, in an eye with total limbal stem cell deficiency (LSCD) associated with underlying corneal scarring and thinning.

**Presentation of case**: A 59-year-old gentleman had poor visual recovery in the right eye (RE) following accidental carbide blast, 1-year before presenting to us. The visual acuity was counting fingers and clinical examination revealed cicatricial entropion involving the upper eyelid, total LSCD, corneal scarring with a central descemetocele and cataract in the RE. Prior to ocular surface reconstruction, entropion correction was performed. Three months later, penetrating keratoplasty combined with cataract surgery and intraocular lens implantation (penetrating keratoplasty (PK) triple), with autologous simple limbal epithelial transplantation (SLET) was performed. The visual acuity was 20/100, 18 months after the surgery, with a clear well-epithelized corneal graft and stable ocular surface.

**Discussion**: LSCD is caused by a decrease in the population and /or function of the limbal epithelial stem cells. Limbal stem cell transplantation (LSCT) is warranted in eyes with total LSCD. In eyes with coexisting corneal scarring, LSCT alone may be inadequate to restore the vision. These eyes require simultaneous or sequential lamellar or full-thickness corneal transplantation for visual rehabilitation. Though, the existing literature favors a sequential approach, where LSCT is performed first followed by corneal transplantation, under certain circumstances such as a thin underlying cornea like in our case, corneal transplantation may have to be combined with LSCT to achieve optimal outcomes.

**Conclusion**: Combining autologous SLET with PK can be performed for visual rehabilitation in eyes with unilateral total LSCD and underlying corneal thinning. Corneal and limbal graft survival is prolonged if existing adnexal comorbidities are addressed before any surgical intervention is planned and adequate time interval is allowed for the surface inflammation to subside.

## Introduction

Ocular chemical injuries are often associated with poor long-term visual outcomes due to chronic sequelae such as limbal stem cell deficiency (LSCD), corneal scarring, and vascularization. ^1^ Visual rehabilitation in these eyes is extremely challenging. To treat LSCD, limbal stem cell transplantation (LSCT) is required. This procedure can be autologous in cases of unilateral LSCD where the limbal epithelial stem cells (LESCs) are harvested from the contralateral eye or this can be allogeneic where the LESCs are harvested from a living-related donor or a cadaveric cornea. ^2^^–^^6^ In eyes with total LSCD and underlying clear corneal stroma, only performing LSCT may stabilize the ocular surface and provide adequate visual outcomes. ^7^ However, in eyes with underlying full-thickness corneal scarring, performing only LSCT may not be sufficient to achieve optimal functional outcomes. In such eyes, a deep anterior lamellar keratoplasty (DALK) or a penetrating keratoplasty (PK) is warranted to restore the corneal transparency. ^8^ However, in the presence of LSCD, performing a PK or DALK alone is known to have very poor outcomes with a higher risk of graft rejection and failure associated with ocular surface inflammation causing persistent epithelial defects. ^9^ In such eyes, optimal visual recovery can be achieved by performing a corneal transplantation either simultaneously with LSCT or sequentially after LSCT.

The published literature recommends that a corneal transplant should be performed once the surface inflammation has decreased, the corneal epithelium is stable and regular, and there is regression of stromal vascularization. ^2^^,^^10^ Poor outcomes have been documented with simultaneous PK and LSCT, with the corneal graft survival ranging from 35% to 64%. ^11^^,^^12^ At present, there are no existing guidelines supporting the relative timing of the two transplant procedures and whether they can be combined as a single stage procedure. In certain cases, performing LSCT and PK becomes inevitable as LSCT alone cannot be performed due to residual corneal thickness being inadequate. We report a case where PK was performed simultaneously with autologous LSCT in an eye with total unilateral LSCD with descemetocele with surrounding corneal scarring and cataract.

## Case presentation

**Figure 1.**
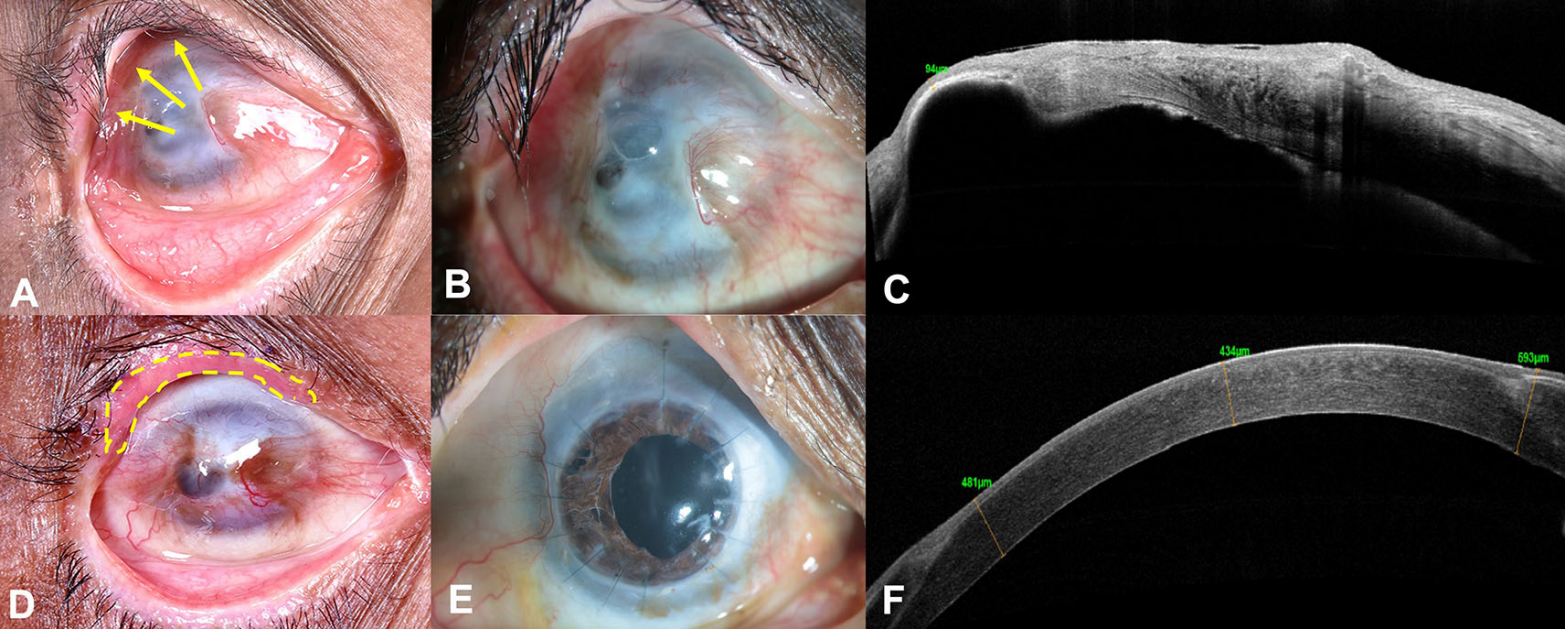
External photograph of the right eye at presentation, shows cicatricial entropion involving the upper eyelid (yellow arrows, A). Slit-lamp image shows diffuse conjunctival hyperemia, pseudopterygium involving two clock hours of the nasal limbus and five clock hours of the temporal limbus with peripheral corneal vascularization involving all quadrants and central descemetocele measuring 3×4 mm (B). Corresponding anterior-segment optical coherence tomography (AS-OCT) image shows dense corneal scarring and severe thinning, with a residual thickness of 94 microns in the area of the descemetocele (C). Three months postoperative image, after entropion correction showing well integrated lid margin mucous membrane graft along the upper eyelid margin (yellow doted lines, D). 18 months postoperative slit-lamp image, after combined autologous simple limbal epithelial transplantation, penetrating keratoplasty triple procedure, showing a well epithelized corneal surface with a clear corneal graft (E). Corresponding AS-OCT image of the same visit, showing compact cornea and corneal epithelium noted over the entire cornea (F).

A 59-year-old Asian Indian male, a farmer by occupation, presented with gradual loss of vision in the right eye (RE) over a period of 1 year, associated with pain, watering and redness following accidental carbide blast. In the acute phase, amniotic membrane transplantation (AMT) was performed twice by the primary ophthalmologist. At presentation, the best corrected visual acuity (BCVA) was counting fingers close to face (CFCF) and 20/20 in the right and left eye (LE), respectively. On clinical examination, the RE showed cicatricial entropion involving the upper eyelid ( Figure 1A, yellow arrows), diffuse conjunctival hyperemia, pseudopterygium involving two clock hours of the nasal limbus and five clock hours of the temporal limbus with peripheral corneal vascularization involving all quadrants ( Figure 1B). Underlying leucomatous corneal scarring was seen with a central descemetocele measuring around 3×4 mm ( Figure 1B). Anterior chamber examination was suggestive of a total cataract, although the details could not be clearly seen. The LE was unremarkable on clinical examination. The anterior segment optical coherence tomography (AS-OCT) of the RE showed corneal thickness of 94 microns in the area of the descemetocele, with surrounding corneal thinning and dense leucomatous corneal scarring ( Figure 1C). Ultrasound B scan showed no obvious abnormality of the posterior segment. The Schirmer’s test revealed a wet ocular surface. Based on the clinical history and examination, a diagnosis of upper eyelid cicatricial entropion, total LSCD, central descemetocele, with full-thickness corneal scar and total cataract was established in the RE.

Prior to performing any surface reconstruction, entropion correction of the upper eyelid with mucous membrane grafting was performed in the RE ( Figure 1D, yellow dotted lines). No recurrence was noted three months post-surgery. Three months following entropion correction, PK triple (PK with cataract extraction and intraocular lens implantation) and autologous SLET was performed with one clock hour of limbus harvested from the superior limbus of the contralateral eye. Pannus dissection was first performed nasally and temporally, and this conjunctiva was allowed to recess from over the cornea. After PK was performed with cataract extraction and lens implantation, and all sutures were placed, an amniotic membrane graft (AMG; Ramayamma International Eye Bank, Hyderabad, India) was secured to the perilimbal sclera with episcleral bites with 10-0 nylon suture and fibrin glue (Tisseel Kit, Baxter AG, Vienna, Austria). The limbal biopsy was cut into multiple pieces and placed on the host cornea peripheral to the graft over the AMG and secured with fibrin glue. A central opening was made in the amniotic membrane. ^13^ A bandage contact lens was placed over the corneal surface. Lateral paramedian permanent tarsorrhaphy was performed to promote epithelization and to protect the ocular surface. Postoperatively, prednisolone acetate (1%) eye drops 1 hourly and moxifloxacin eye drops (0.5%) four times daily were prescribed. After 1 week, antibiotic eye drop was discontinued and topical steroid drop was tapered gradually over the subsequent weeks to four times daily after 6 weeks, which was continued for 6 months of follow-up after which this was further stepped down to thrice daily.

After complete epithelization of the ocular surface, the tarsorrhaphy was released. On further follow-ups post-surgery, raised intra-ocular pressure was documented with cup-to-disc ratio (CDR) of 0.8:1. A diagnosis of secondary glaucoma was established, and the patient has been managed on topical anti-glaucoma medications (combination of timolol maleate 0.5% and brimonidine tartrate 0.2% twice daily, dorzolamide 2% thrice daily) with good intra-ocular pressure control until the last follow-up visit. Patient was also started on topical cyclosporine 0.1% two times a day in view of having a high-risk graft. The patient has completed 18 months of postoperative follow-up and maintains a BCVA of 20/100 in the RE. Postoperatively, the graft is clear and well epithelized, with a stable ocular surface ( Figure 1E). The AS-OCT of the RE revealed a compact cornea with a central corneal thickness of 434 microns and uniform corneal epithelium ( Figure 1F). The LE only had discontinuity at the limbus at the site of limbal biopsy, otherwise the cornea was well epithelized and clear.

## Discussion

The outcome of a corneal or limbal transplant is determined by presence of comorbidities such as tear film abnormalities, lid and lid margin related disease, uncontrolled intraocular pressure and presence of active surface inflammation. ^10^ To reduce the risk of graft rejection and failure, it is important to address these existing comorbidities prior to performing any transplant procedure. ^9^ In our case, prior to performing ocular surface reconstruction for visual rehabilitation, entropion correction with mucous membrane grafting was performed. This procedure of entropion correction with anterior lamellar recession and labial mucous membrane graft (MMG) is a well-established technique with modifications in the suturing technique. The risk of surgical failure increases in eyes with cicatricial entropion secondary to non-infectious etiology such as Stevens–Johnson syndrome, burns, and trauma. ^14^ The modified technique used in this patient, has been described by Singh *et al.*, where labial MMG is used for spacing the anterior lamella and reconstruction of the lid margin and the posterior lamella. ^15^ This technique has shown promising long-term outcomes, particularly in eyes with cicatricial entropion secondary to ocular surface cicatricial disorders.

Over several years, conjunctival limbal autograft (CLAU), cultivated limbal epithelial transplantation (CLET), keratolimbal allograft (KLAL) have been successfully performed in eyes with LSCD. ^2^^,^^12^^,^^16^ However, limitations exist with different procedures such as the risk of iatrogenic LSCD in the contralateral healthy donor eye with CLAU, difficulty in the surgical procedure with the need for life long systemic immunosuppression and need for a donor cornea with KLAL, and the need for an expensive laboratory setting to allow *ex vivo* expansion of LESCs in CLET. ^17^ Autologous simple limbal epithelial transplantation (SLET) introduced in 2012, has quickly gained popularity as it uses minimal limbal tissue from the contralateral healthy eye, allows *in vivo* expansion of stem cells without the risk of iatrogenic LSCD in the donor eye, and also alleviates the need for systemic immunosuppression. ^6^ However, in eyes with LSCD and corneal scarring, the results of LSCT procedures combined with PK have not been encouraging. Shimazaki *et al.* and Solomon *et al.* published outcomes of simultaneous KLAL and PK in eyes with total LSCD. ^11^^,^^18^ Inferior outcomes were seen in eyes where simultaneous procedure was performed as compared to those eyes where two-staged surgery was performed (KLAL followed by PK) with higher rate of corneal graft rejection. Corneal graft rejection has been attributed to increased exposure of the host immune system to the donor corneal antigens through the limbal allograft antigens, which are derived from the same donor as the central graft. Also, simultaneous PK increases the surface inflammation and the wound healing response. Both studies recommended a time interval of 3–6 months after KLAL to allow stabilization of the ocular surface before performing keratoplasty. ^11^^,^^18^

In 2011, Basu *et al.* compared anatomical and functional outcomes of simultaneous PK and CLET with two-staged procedure in eyes with unilateral total LSCD secondary to ocular burns. ^19^ They concluded that a two-staged surgical approach of first transplanting the cultivated limbal stem cells, followed by PK, resulted in superior outcomes in restoring the ocular surface stability and vision, as compared to single-staged procedure. Also, once the surface was well epithelized, which usually takes around 6 weeks, the time interval between CLET and PK did not affect the corneal graft survival. This was in accordance with the outcomes published by Baradaran-Rafii *et al.* and Sangwan *et al.*, where a two-staged sequential surgical approach showed favorable outcomes in terms of corneal graft survival and final visual acuity, as compared to simultaneous surgeries. ^20^^,^^21^

The existing literature reporting outcomes of simultaneous autologous SLET and PK have also been discouraging. Simultaneous PK is an established risk factor for recurrence of LSCD and failure of SLET in eyes with total unilateral LSCD in adults and children. ^8^^,^^22^ The cause for poor visual recovery in eyes is complications like secondary glaucoma, amblyopia and infectious keratitis, rather than the surgical procedure itself. Encouraging outcomes have been reported in eyes where sequential ocular surface reconstruction by SLET followed by keratoplasty was performed in eyes with unilateral total LSCD. ^23^^,^^24^ Simultaneous allogeneic SLET with PK has been described in a case of sterile keratolysis following severe ocular chemical burn, as an emergency procedure to preserve the globe tectonicity. ^25^

Surgical decision making in such cases depends on the status of the contralateral eye. Of the various techniques described earlier for ocular surface reconstruction, in our case, autologous SLET was preferred over CLAU, CLET or allogeneic SLET. The need for a CAG or CLAU was eliminated in our case as there was no co-existing symblepharon. As our patient had a healthy contralateral eye, autologous LSCT was preferred to avoid the need for systemic immunosuppression. Similarly, for visual rehabilitation, PK was preferred over Boston type 1 keratoprosthesis (BKpro1), which is often reserved in patients who are bilaterally blind. Though BKpro1 has shown to provide rapid visual recovery in eyes with LSCD secondary to chemical burns, glaucomatous optic neuropathy is common in these eyes and may reduce the long-term visual potential post BKpro1. ^26^ Also, when BKpro1 is performed in a patient with a contralateral healthy eye, these patients may not be aware of the decreased vision in the operated eye, secondary to any complication, as the contralateral eye maintains a good vision. ^27^

Hence, in our case, we chose to perform autologous SLET combined with PK. Although sequential surgery would have been ideal, this was not possible in our case due to the underlying cornea being thin. Corneal thinning increases the risk of frank perforation of the cornea during pannus dissection for SLET, thus necessitating PK. Thus, performing SLET alone was not a possibility. Although performing PK alone could have been a possibility, this would have led to recurrence of LSCD over the graft, thus, leading to failure of the PK procedure. Also, in our case, there were certain modifications to the surgical procedure. Pannus dissection was not performed 360 degrees, as is performed in SLET as a complete pannus was not seen over the cornea. Hence pannus dissection was performed only in the areas of the pannus nasally and temporally and this conjunctiva was allowed to recess. Sutures were used to anchor the AMG on the perilimbal sclera as the underlying cornea was epithelized and it is possible that the AMG would not have been stable otherwise. Also, as the pannus dissection was not performed 360 degrees, it was not possible to tuck the AMG under the surrounding conjunctiva, as is performed in SLET. Hence, sutures were deemed to be necessary. Additionally, while performing SLET, the explants were placed peripherally over the host cornea. This surgical modification can prevent the loss of explants, in eyes where a repeat penetrating keratoplasty is required. Also, in these cases, it may be more important to support the paracentral area and the limbal area in the form of a doughnut AMG. ^13^ The central area of the corneal graft may have healthy donor corneal epithelium which need not be debrided. Hence, AMG may not be required centrally, and in our case, a central opening was created in the AMG, thus allowing for better visual acuity. ^13^ Also, it has been reported that AMG may persist as a subepithelial membrane in some cases leading to haze and poor visual acuity post-operatively. ^28^ This central opening is not created in SLET cases as the underlying cornea is devoid of epithelium and if the AMG does not cover this area, then this area of the corneal stroma may not get epithelized as there is no continuous scaffold to grow over.

An allogeneic SLET was not performed as the recipient will then be exposed to antigens from two donors versus a single donor. ^10^ Another option would be choosing to take a larger graft and include the limbus from the donor graft, however, this would also require systemic immunosuppression. ^29^ Simultaneous intervention in our case also allowed for faster visual recovery, reduced multiple follow-up visits for the patient, reduced the cost of surgery, and allowed for an earlier diagnosis and management of secondary glaucoma. We also believe that addressing the adnexal abnormalities prior to ocular surface reconstruction helped us achieve the desired outcome of a clear graft with a stable ocular surface which was maintained for a follow-up period of 18 months.

## Consent

Written informed consent for publication of their clinical details and clinical images was obtained from the patient.

## Data Availability

All data underlying the results are available as part of the article and no additional source data are required. Mendeley: CARE checklist for ‘Case Report: Simultaneous penetrating keratoplasty with autologous simple limbal epithelial transplantation as an alternative to keratoprosthesis’. https://doi.org/10.17632/m299chb22v.2 Data are available under the terms of the Creative Commons Zero “No rights reserved” data waiver (CC0 1.0 Public domain dedication).
