# Supplementary material for: Case Report: Simultaneous penetrating keratoplasty with autologous simple limbal epithelial transplantation as an alternative to keratoprosthesis
Source: F1000Res. 2023 Jul 20;12:488. Originally published 2023 May 12. [Version 3] doi: 10.12688/f1000research.133637.3 (PMC10349272; doi:10.12688/f1000research.133637.3)
Supplement: Peer review history

# Peer review: Reviewer response for version 2

**DOI:** 10.5256/f1000research.152953.r187446

**Published:** 2023-07-14

**Authors:** Sotiria Palioura

**Affiliations:** 1 Department of Ophthalmology, University of Cyprus Medical School, Aglantzia, Cyprus

The authors have addressed adequately all the comments. Congratulations for successful management of this challenging case!

Are enough details provided of any physical examination and diagnostic tests, treatment given and outcomes?

Yes

Is the case presented with sufficient detail to be useful for other practitioners?

Partly

Is sufficient discussion included of the importance of the findings and their relevance to future understanding of disease processes, diagnosis or treatment?

Yes

Is the background of the case’s history and progression described in sufficient detail?

Yes

Reviewer Expertise:

Cornea

I confirm that I have read this submission and believe that I have an appropriate level of expertise to confirm that it is of an acceptable scientific standard.

# Peer review: Reviewer response for version 1

**DOI:** 10.5256/f1000research.146644.r180027

**Published:** 2023-07-10

**Authors:** Denise Loya Garcia

**Affiliations:** 1 Instituto de Ciencias Visuales y Oftalmología, Hospital Zambrano Hellion, San Pedro Garza García, Mexico

The authors present a very interesting case highlighting the advantages of autologous simple limbal epithelial transplantation combined with penetrating keratoplasty as a single-stage procedure.

 Despite the evidence supporting a two-stage procedure, the authors clearly explained and justified the use of combined surgery by a multidisciplinary team and an organized surgical approach in a well-selected patient such as the one described in this case report. Underscoring the importance of addressing the adnexal abnormalities before any ocular surface reconstruction is key.

 The authors used the “Doughnut” amniotic membrane transplantation, my suggestion is that they could explain the benefits of using this technique for retaining a clear central cornea after AMT transplantation.

Are enough details provided of any physical examination and diagnostic tests, treatment given and outcomes?

Yes

Is the case presented with sufficient detail to be useful for other practitioners?

Partly

Is sufficient discussion included of the importance of the findings and their relevance to future understanding of disease processes, diagnosis or treatment?

Yes

Is the background of the case’s history and progression described in sufficient detail?

Yes

Reviewer Expertise:

Cornea, Ocular Surface, Dry Eye.

I confirm that I have read this submission and believe that I have an appropriate level of expertise to confirm that it is of an acceptable scientific standard.

# Peer review: Reviewer response for version 1

**DOI:** 10.5256/f1000research.146644.r173526

**Published:** 2023-05-25

**Authors:** Sotiria Palioura

**Affiliations:** 1 Department of Ophthalmology, University of Cyprus Medical School, Aglantzia, Cyprus

This case report nicely documents the sequence of rehabilitative procedures that need to be done in order to restore vision in an eye with cicatricial entropion, corneal thinning, cataract and total LSCD following thermal injury. They should be congratulated for a great outcome on an eye that would otherwise be lost.

 Although the cited literature suggests that a two stage procedure has better long-term outcomes in terms of graft survival, in certain cases, such as the one described by the authors, this cannot be done due to the significant corneal thinning and the risk of perforation during pannus dissection for the LSCT.

 The authors should discuss in more detail why corneal thinning precludes a sequential procedure. Moreover, they should discuss how they modified their technique. It seems that they did not perform a 360 peritomy as is commonly done in SLET and pannus dissection, but rather performed the PK and then secured the AMT over the peripheral conjunctiva.

 Finally, the authors should discuss why they performed a central opening in the ΑΜΤ and whether, in their experience, this leads to better visual quality in the long term. Why did they choose to do that in this case? Or is it something they always perform in their SLET procedures?

Are enough details provided of any physical examination and diagnostic tests, treatment given and outcomes?

Yes

Is the case presented with sufficient detail to be useful for other practitioners?

Partly

Is sufficient discussion included of the importance of the findings and their relevance to future understanding of disease processes, diagnosis or treatment?

Yes

Is the background of the case’s history and progression described in sufficient detail?

Yes

Reviewer Expertise:

Cornea

I confirm that I have read this submission and believe that I have an appropriate level of expertise to confirm that it is of an acceptable scientific standard, however I have significant reservations, as outlined above.
